# Vortioxetine: A Potential Drug for Repurposing for Glioblastoma Treatment via a Microsphere Local Delivery System

**DOI:** 10.1021/acsbiomaterials.5c00068

**Published:** 2025-04-01

**Authors:** Yu Wang, Dorit Siebzehnrubl, Michael Weller, Tobias Weiss, Florian A. Siebzehnrubl, Ben Newland

**Affiliations:** † School of Pharmacy and Pharmaceutical Sciences, Cardiff University, King Edward VII Avenue, Cardiff CF10 3NB, United Kingdom; ‡ Cardiff University School of Biosciences, European Cancer Stem Cell Research Institute, Cardiff CF24 4HQ, United Kingdom; § Department of Neurology, Clinical Neuroscience Center, University Hospital Zurich and University of Zurich, Zurich 8091, Switzerland

**Keywords:** glioblastoma, droplet-based microfluidic, oil-in-oil emulsion, drug repurposing, PLGA

## Abstract

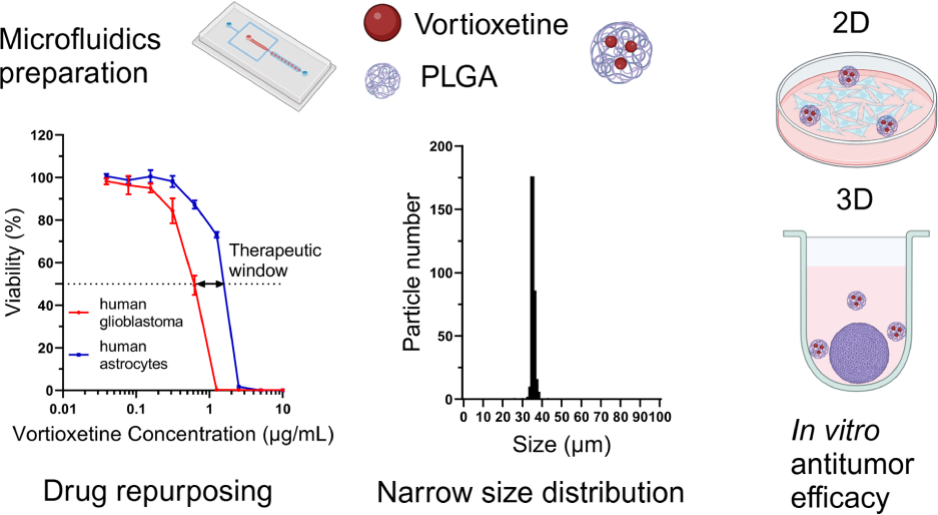

Drug repurposing is an attractive route for finding new therapeutics for brain cancers such as glioblastoma. Local administration of drugs to brain tumors or the postsurgical resection cavity holds promise to deliver a high dose to the target site with minimal off-target effects. Drug delivery systems aim to sustain the release of the drug at the target site but typically exhibit drawbacks such as a poor safety profile, uncontrolled/rapid drug release, or poor control over synthesis parameters/material dimensions. Herein, we analyzed the antidepressant vortioxetine and showed *in vitro* that it causes a greater loss of viability in glioblastoma cells than it does to normal primary human astrocytes. We developed a new droplet microfluidic-based emulsion method to reproducibly produce vortioxetine-loaded poly(lactic-*co*-glycolic) acid (PLGA) microspheres with tight size control (36.80 ± 1.96 μm). The drug loading efficiency was around 90% when 9.1% (w/w) drug was loaded into the microspheres, and drug release could be sustained for three to 4 weeks. The vortioxetine microspheres showed robust antiglioblastoma efficacy in both 2D monolayer and 3D spheroid patient-derived glioblastoma cells, highlighting the potential of combining an antidepressant with sustained local delivery as a new therapeutic strategy.

## 1 Introduction

Glioblastoma (GBM) is the most malignant primary brain cancer, associated with poor clinical outcomes and high mortality.^1^ The median survival time after the first diagnosis is less than two years.^2,3^ The standard of care, established in 2005, is still the routine treatment for GBM and includes maximal safe resection (when possible/practical) followed by radiotherapy plus adjuvant Temozolomide chemotherapy.^4^ However, half of GBM patients are resistant to Temozolomide, which has been linked to promoter methylation of O^6-^methylguanine-DNA methyltransferase (MGMT), a DNA damage repair protein.^5^ Bevacizumab was approved by the Food and Drug Administration (FDA) to treat recurrent GBM but has thus far failed to show a benefit in overall survival time.^6,7^ Lomustine, an alternative chemotherapeutic, has also not shown significant advantages over Temozolomide when the drug was used alone in a randomized controlled trial.^8−10^ There is, therefore, an urgent need to find effective, safe, and long-lasting therapeutic agents for GBM.

At present, synthesizing novel active ingredients is becoming increasingly challenging. The burden of investment to find the next generation of chemotherapeutics is growing, fueling the rationale for drug repurposing approaches.^11^ By repurposing or repositioning existing drugs for new indications, the time and money invested per successful outcome can potentially be reduced.^12^ There are dozens of FDA-approved drugs that have proven activity in GBM models, with some of them being tested in clinical trials.^13−16^

We previously screened 67 repurposed neuroactive drugs in 27 *ex vivo* GBM patient samples.^17^ The resulting pharmacoscopy score, which was quantified by measuring the changes in cell population fraction, showed that vortioxetine scored the highest and had a high specificity for killing intertumor and intratumor heterogeneous GBM cells. In addition, vortioxetine also gave a significant survival benefit in comparison to the vehicle control in an orthotopic xenograft GBM mouse model and a survival benefit comparable to that of Temozolomide treatment. Vortioxetine is a 5-hydroxytryptamine receptor antagonist used to treat major depressive disorder, approved by the FDA in 2013.^18^ It has a high fraction of plasma protein binding, with 99% of the drug being protein bound, suggesting limited free drug availability for a therapeutic effect.^19^ With little else known about its potential as a GBM therapeutic, we hypothesized that local delivery to the tumor may increase its antitumor efficacy.

Since surgical resection of the tumor is applicable to most GBM patients, local delivery into the resection cavity is an attractive strategy to circumvent the blood-brain barrier (BBB), achieve high drug concentrations at the residual tumor site, and reduce systemic side effects. The Gliadel wafer is the only FDA-approved local treatment drug for GBM. However, the survival benefit for patients treated with Gliadel has been modest.^20^ A systematic review revealed that the median overall survival of patients who received Gliadel plus radiotherapy and TMZ was 18.2 months,^21^ compared to 14.6 months after the standard of care.^4^ Additionally, the large, stiff wafer needs gross tumor debulking to get enough space for implantation and has been associated with side effects when dislodged.^22,23^ Injectable drug delivery systems offer additional flexibility in terms of application, either through use in uneven/small cavities, or via methods, such as convection-enhanced delivery to otherwise inoperable tumors.^24^ Much research has focused on the development of hydrogel, nanoparticle, and microparticle delivery systems,^25^ but the goal of a nontoxic, well-defined, and reproducible drug delivery system, suitable for regulatory approval with a slow drug release profile, has thus far remained elusive.^26^

Microspheres, which are defined as particles with a size range between 1 and 1000 μm, can encapsulate drugs within their homogeneous matrix as single molecules or small clusters.^27^ Microspheres smaller than 250 μm can be considered as injectable preparations depending on the cannula used.^28^ Poly(d,l-lactide-*co*-glycolide) (PLGA) is a biocompatible and biodegradable lactic acid and glycolic acid copolymer approved by the FDA for clinical use.^29^ PLGA was chosen over other polymers for simplicity in design, with controllable degradation properties and good solubility in numerous organic solvents, making it an attractive starting point for delivery system synthesis.^30^

Many methods can be used to prepare PLGA microspheres, including double or multiple emulsion solvent evaporation,^31,32^ cryogenic solvent extraction,^33^ catalytic hydrolysis solvent removal,^34^ nonsolvent addition,^35^ spray-drying,^36^ supercritical fluids,^37^ and membrane emulsification.^38^ However, the size distribution of PLGA microspheres is typically very poor. For example, the commonly used emulsion solvent evaporation method relies on nonuniform mechanical forces to create droplets, resulting in high size dispersity.^39^ Polydispersity reduces reproducibility and introduces variation, as particle size affects factors such as drug release and the degradation rate. Factors such as the drug molecular distribution in the microspheres, surface area-to-volume ratio, and porosity all affect polymer hydrolysis and drug dissolution.^40,41^ Tight control over these factors is essential for producing reproducibly efficacious and regulatory-approved therapeutics. A new approach to preparing monodisperse PLGA microspheres with a smooth surface (low porosity) and a regular round shape is therefore desirable.

We aimed to validate vortioxetine as an antiglioblastoma therapeutic and to combine droplet-based microfluidics with a new emulsion formula, to create vortioxetine-loaded, monodispersed PLGA microspheres as a locally administered sustained therapeutic for GBM. Herein, we showed that free vortioxetine was more toxic toward patient-derived GBM cell lines than to primary human astrocytes, indicating a potential therapeutic window minimizing the side effects to healthy cells. Then, a water-free oil-in-oil (O/O) emulsion was created in a microfluidic device to prepare empty and vortioxetine-loaded microspheres (termed vortioxetine microspheres) with high reproducibility and monodispersity. The cytocompatibility of the empty PLGA microspheres was demonstrated *in vitro* on human astrocytes, and sustained drug release from vortioxetine microspheres effectively killed patient-derived GBM cells both in 2D culture and in 3D tumor spheroids.

## 2 Materials and Methods

### 2.1 Chemicals and Materials

The following chemicals were purchased from Merck: Resomer RG 752 H, poly(d,l-lactide-*co*-glycolide) (PLGA, L:G 75:25, *M*_w_: 4000–15000, 719919), 1H,1H,2H,2H-perfluoro-1-octanol (PFO, 370533), eosin Y (E6003), sodium acetate trihydrate (32318), acetic acid (27225), fetal bovine serum (FBS) (F7524), penicillin-streptomycin (P4333), 0.25% trypsin-EDTA solution (T4049), poly-l-lysine (P6282), transferrin (T8158), putrescine (P5780), sodium selenite (S5261), progesterone (P8783), insulin (I5500), hydrochloric acid solution (H9892), heparin (H4784), and DMSO (D2650). The following solvents were purchased from Fisher Chemical: absolute ethanol (E/0600DF/15), acetonitrile (A/0632/PB15), water with 0.1% formic acid (v/v) (10229884), and acetonitrile with 0.1% formic acid (v/v) (10678935).

Phosphate-buffered saline (PBS, 10010023), DMEM/F12 with GlutaMAX (10565018), and DMEM/F12 (1:1, 21331046) were purchased from Gibco. HFE-7500 3M Engineered Fluid (7100025016) was purchased from 3M. FluoroSurfactant 008 (038–074) was purchased from RAN Biotechnologies. Vortioxetine (ABIN6574672) was purchased from TargetMol. MycoZap Plus-CL (VZA-2011) was purchased from Lonza. FGF2-G3 (Qk053) was purchased from Qkine. Accutase (00–4555–56) and Geltrex Basement Membrane Matrix (A1413202) were purchased from Thermo Fisher. PrestoBlue (A13261) was purchased from Invitrogen. AF rhEGF (AF-100–15) was purchased from PeproTech.

### 2.2 Preparation of PLGA Microspheres

A droplet-based microfluidic technique was used to create PLGA microspheres. A cross-junction configuration microfluidic chip with an 80 μm nozzle size was used (Microfluidic Chip Fluidic 440, Microfluidic ChipShop, Germany). The continuous phase contained 2% 008 Fluoro Surfactant in HFE-7500 oil, while 100 mg/mL PLGA and 10 mg/mL vortioxetine were dissolved in acetonitrile as the dispersed phase. To prepare empty PLGA microspheres, the dispersed phase contained only 100 mg/mL PLGA. The flow rates of the continuous phase and the disperse phase were set at 900 and 150 μL/h, respectively (driven by the LA30 syringe pump, Imprint Landgraf Laborsysteme HLL GmbH, Germany). An inverted microscope (491206–0002–000, Zeiss, Germany) with a high-speed camera (C110, Vision Research Ltd., UK) was used to monitor droplet generation. After the droplet generation was stable, droplets were collected in 2 mL Eppendorf tubes. To purify the PLGA microspheres, the droplets were dried in a vacuum oven (OVL-570–010J, Gallenkamp, UK) at room temperature for 3 h to remove the acetonitrile. The HFE oil was aspirated from the bottom of the tube. Microspheres were washed three times using 100 μL of 20% (v/v) PFO in HFE and three times with 200 μL of HFE oil. The PLGA microspheres were dried in a vacuum oven at room temperature overnight to remove the remaining HFE oil.

### 2.3 Morphological Characterization of PLGA Microspheres

#### 2.3.1 Bright Field Microscope Images

Three batches of vortioxetine microspheres were prepared to evaluate their reproducibility. Dried microspheres were resuspended in PBS. The size of 300 microspheres was analyzed for each batch. The size of the microspheres was measured by the particle analysis function in ImageJ, and the diameter of the microspheres was calculated by the circle area formula.

#### 2.3.2 Scanning Electron Microscope (SEM)

The morphology of microspheres was visualized by a Zeiss Sigma HD Field Emission Gun Scanning Electron Microscope (Zeiss, Germany). To prepare the samples, the microspheres were coated with AuPd using a BIO-RAD SC500 sputter coater (Quorum Technologies, UK). The microspheres were uniformly covered with a thick layer of AuPd at around 10–20 nm. A beam energy of 5 kV with a 30 μm diameter final aperture was used, and the microspheres were imaged via an Everhart-Thornley detector.

### 2.4 Fourier Transform Infrared Spectroscopy (FTIR)

Infrared spectra of PLGA, free vortioxetine, and vortioxetine microspheres were scanned by the IRSpirit FTIR Spectrometer (Shimadzu Co., Ltd., Japan) using dried powder samples directly. The background signal was scanned before running each sample to subtract any residual peaks from the instrument and the environment. FTIR spectra were scanned in transmittance mode from 500 cm^–1^ to 3500 cm^–1^ with a resolution of 0.9.

### 2.5 Analysis of Drug Loading Efficiency

Ultraperformance liquid chromatography (ACQUITY UPLC System, Waters, USA) was used to detect vortioxetine for the drug loading efficiency study. A photodiode array (PDA) detector was used, and the PDA spectrum between 253 and 600 nm was used to quantify the concentration of the drug. The mass spectrometry (MS) detector was used to confirm the peak in the PDA spectrum belonging to vortioxetine. The calibration curve was built by preparing 1, 2, 4, 6, 8, and 10 μg/mL drug solutions. To investigate the loading efficiency, vortioxetine microspheres with five different weight ratios of drug to PLGA (1:2, 1:3, 1:4, 1:5, and 1:10) were prepared. The drug concentration in the dispersed phase was kept constant at 10 mg/mL, and the weight ratio was changed by adjusting the PLGA concentration in the dispersed phase. One mg of vortioxetine microspheres was weighed, dissolved in 1 mL of acetonitrile, and diluted to a suitable concentration within the linear range of the calibration curve. The drug loading efficiency was calculated by the following equation (eq 1):
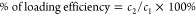
1

where *c*_*1*_ is the theoretical drug concentration in the samples and *c*_*2*_ is the actual concentration in the samples.

### 2.6 Evaluation of *In Vitro* Drug Release Profiles

Pure PBS was used as the release medium to mimic physiological conditions. Vortioxetine microspheres (1:10 weight ratio of drug to PLGA), containing 50, 100, 300, or 500 μg of drug, were incubated in an incubator (Orbital Shaker Incubator, Grant-bio, UK) at 37 °C with gentle shaking at 80 rpm. At each time point, after centrifugation, 900 μL of supernatant was removed, stored, and replaced with fresh release medium. Microspheres were resuspended by vortex mixing. The samples were stored at −20 °C for further investigation. At the end of the experiment, the concentration of the drug in all samples was detected by a fluorescence quenching method.^42^ Briefly, 300 μL of samples, 300 μL of 0.15 mg/mL eosin Y, and 300 μL of 0.2 M acetic acid buffer at pH 3.7 were added into a 2 mL Eppendorf tube and mixed well by a vortex mixer. The fluorescence intensity of eosin Y was read using excitation and emission wavelengths of 306 and 539 nm, respectively. The calibration curve method was used to quantify the drug concentration (linear range of 0.4 to 8 μg/mL). The cumulative amount of released drug was calculated by the following equation (eq 2):
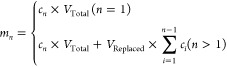
2

where *m*_*n*_ is the cumulative release amount at the *n*th time point (μg), *c*_*n*_ is the drug concentration at the *n*th time point (μg/mL), *V*_Total_ is the total volume of the release medium, and *V*_Replaced_ is the volume of the replaced release medium at each time point.

### 2.7 Cell Culture

#### 2.7.1 Primary Human Astrocytes

Primary human astrocytes, isolated from the cerebral cortex, were purchased from ScienCell Ltd. (USA). Cells were cultured as instructed by the manufacturer, with a slight modification, which was 10% FBS in DMEM/F12 being used instead of the trypsin/EDTA neutralization solution available from the manufacturer. Briefly, the astrocytes were cultured in astrocyte medium (1801, ScienCell Ltd., USA) in a 37 °C, 5% CO_2_ incubator. Cell culture flasks were coated with 2 μg/mL poly-l-lysine. The cell culture medium was changed every 2 to 3 days until confluency reached 90–95%. Cells were dissociated using a 0.025% trypsin-EDTA solution at room temperature. Cells were plated at a density of 5,000 cells/cm^2^.

#### 2.7.2 Human GBM Cell Lines

Patient-derived GBM cell lines (hGBM L0, L1, and L2) were cultured as previously reported.^43,44^ Briefly, hGBM cells L0, L1, and L2 were grown as suspension cells in N2 medium with 20 ng/mL of rhEGF and 3 ng/mL of thermostable rhFGF2 G3 in a 37 °C, 5% CO_2_ incubator. Typically, cells were subcultured every week. Cells were dissociated using Accutase and counted using a Beckman Coulter Z2 (Beckman Coulter Inc., USA). Cells were plated at a density of 10,000 cells/cm^2^ to account for their slow growth rate.

N2 medium was prepared by adding 1 mL of MycoZap Plus-CL and 500 μL each of Transferrin-Putrescine-Sodium Selenite (TPN), insulin, and progesterone to 497.5 mL of DMEM/F12 (Glutamax). TPN was prepared by dissolving 500 mg of transferrin and 81 mg of putrescine in deionized water and adding 25 μL of sodium selenite stock solution (10.5 mg of sodium selenite was dissolved in 10 mL of deionized water) to a final volume of 5 mL. Insulin solution was prepared by dissolving 25 mg of insulin in 5 mL of 0.1 M HCl. 6.3 mg of progesterone was dissolved in 10 mL of absolute ethanol to prepare the stock solution, and 50 μL of the stock solution was diluted to a 5 mL solution with deionized water to get the final progesterone solution. TPN, insulin, and progesterone solutions were then filter-sterilized and stored at −20 °C. The feeding solution to support the growth of cells contained 400 ng/mL rhEGF, 60 ng/mL thermostable FGF2 G3, and 40 μg of heparin. When culturing hGBM cells, 50 μL of feeding solution was added to the N2 medium to get 1 mL of complete medium.

Tissue culture plates were coated with a Geltrex basement membrane matrix. The plates were incubated at 37 °C for 1 h and put at room temperature for another 1 h before use. Cells were seeded immediately after aspirating the supernatant of the Geltrex solution.

### 2.8 Cytocompatibility of Empty PLGA Microspheres

The cytocompatibility of empty PLGA microspheres was evaluated by using primary human astrocytes. Cells were seeded in poly-l-lysine-coated 48-well plates (Costar, 3548) at a density of 4,800 cells/well. After 1 day of incubation, empty PLGA microspheres were added to get a final concentration of 100 μg/mL. Images of cells were taken via a microscope camera (A35180U3, OMAX Microscope, South Korea), and cell viability was tested using the PrestoBlue assay on days 1, 4, and 7 after adding PLGA microspheres and normalizing to untreated control cells (background fluorescence was subtracted from experimental wells). Cells were incubated in PrestoBlue for 2 h and the fluorescence intensity was read with an excitation wavelength of 560 nm and an emission wavelength of 590 nm by the bottom-reading model.^45^

### 2.9 Cytotoxicity of Free Vortioxetine

To investigate the cytotoxicity of free vortioxetine, primary human astrocytes were plated in poly-l-lysine-coated 96-well plates (Costar, 3595) at a density of 1,600 cells/well, and hGBM cell lines were plated in Geltrex-coated 96-well plates at a density of 5,000 cells/well. After 1 day of incubation, a vortioxetine solution was added, which was prepared by serial dilution from a 5 mg/mL stock solution in pure DMSO using complete culture medium. Cell viability was tested using the PrestoBlue assay 1, 4, and 7 days after the addition of the drugs to the cells.

### 2.10 Antitumor Efficacy of Vortioxetine Microspheres in 2D Cell Culture Models

The hGBM L0, L1, and L2 cells were cultured in Geltrex-coated 48-well plates as adherent cells to investigate the *in vitro* antitumor efficacy. Cells at a density of 15,000 cells/well were cultured for 1 day, and then drugs were added to get a final concentration of 100 μg/mL for empty PLGA microsphere controls; 0.625, 1.25, 2.5, 5, and 10 μg/mL for vortioxetine microspheres; and 1.25 μg/mL for the free drug control. 1, 4, or 7 days after adding the microspheres/drug, the cells were imaged, and cell viability was analyzed using the PrestoBlue assay.

### 2.11 Analyzing the Efficacy of Vortioxetine Microspheres Against 3D GBM Spheroids

Ultralow attachment 96-well plates (Costar, 7007) were used to establish the 3D spheroid model. Briefly, hGBM L0 and L2 cells were seeded at a density of 1,000 cells/well. The well plates were centrifuged at 300 *g* for 5 min. The spheroids were incubated for 4 days before adding the experimental groups and then treated with 1.25, 2.5, 5, and 10 μg/mL vortioxetine microspheres or 1.25 μg/mL of free drug. Images of spheroids were taken on days 4, 5, 6, 7, 8, 11, 13, 15, and 18. The size of the spheroids was measured using ImageJ. On days 5, 8, 11, and 18, additional cultures of the spheroids were incubated with PrestoBlue for 3 h before reading the fluorescence intensity. To investigate the efficacy of vortioxetine microspheres against larger, pre-established spheroids, hGBM L0 and L2 spheroids were cultured for 7 days before adding the experimental groups. The size of the spheroids was monitored on days 7, 8, 10, 12, 14, and 16, and cell viability was evaluated on days 12 and 16.

### 2.12 Statistical Analysis

The statistical analysis was conducted using GraphPad Prism (8.4.3). First, the assumption of normality was checked by the Shapiro-Wilk test. To compare two groups of data, if both groups of data passed the normality test, Student’s *t*-test was used for data without significant different variances, and Welch’s test was used for data with significant different variances. To compare three or more groups of data, if all groups of data passed the normality test, a one-way ANOVA test was used for data with homogeneity of variances (checked by the Brown-Forsythe test), and Welch’s ANOVA test was used for data with unequal variances. Posthoc tests (Tukey’s multiple comparisons test for ordinary one-way ANOVA test, Dunnett’s T3 multiple comparisons test for Welch’s ANOVA test) were done if the results of the overall ANOVA test were significantly different, to determine which specific group was significantly different from the other group. A two-way ANOVA test was used to analyze 3D cell study results with Tukey’s posthoc test. A *p*-value of ≤0.05 was defined as a significant difference. No significant difference (ns), *p* > 0.05; * denotes *p* ≤ 0.05; ** denotes *p* ≤ 0.01; *** denotes *p* ≤ 0.001; **** denotes *p* ≤ 0.0001.

## 3 Results and Discussion

### 3.1 Preparation and Characterization of PLGA Microspheres

At present, emulsion-solvent evaporation still dominates the preparation of PLGA microspheres either in laboratories or in commercial products. A single oil-in-water (O/W) emulsion is commonly used to encapsulate hydrophobic drugs in PLGA,^31^ while hydrophilic drugs, including proteins, loaded into PLGA microspheres are usually prepared by a water-in-oil-in-water (W/O/W) double emulsion.^46^ Although relatively good control of size distribution has been achieved, it is still challenging to produce high consistency in product sizes. Many factors can affect the production of PLGA microspheres by these methods, including physical parameters such as stirring rate and volume ratio of the two phases, physicochemical parameters, such as viscosity and density, and chemical parameters, such as surfactant and solvent.^47^ Droplet-based microfluidics is an emerging technology to prepare microspheres.^48−50^ The generation of droplets can be monitored in real time, and the size and morphology of microspheres can be precisely controlled by the geometry of the microfluidic device, flow rate ratio, and viscosity of the two phases. One of the drawbacks of microfluidics is its low production efficiency. Using multiple devices or multiple microchannel devices is a simple way to increase production efficiency.^51^ However, a typical PLGA microsphere synthesis protocol using organic solvents, such as dichloromethane, is unsuitable for adaptation to commonly used microfluidic devices made from polydimethylsiloxane (PDMS). The solvent can cause the PDMS to swell, thus changing channel dimensions if not ruining the device. Glass microfluidic devices could potentially be used, but at a high cost further hampering scale-up for production.

Cyclic olefin copolymers (COC) make up a novel class of polymeric materials. The strengths of COC include high transparency, rigidity, strength, hardness, biocompatibility, and very good resistance to acids, alkalis, and polar solvents.^52^ We therefore used microfluidic chips made from COCs to prepare PLGA microspheres. However, these are still not resistant to dichloromethane, so another solvent with a low boiling point (for ease of removal postsynthesis) was sought for use with the devices. The requirements to prepare PLGA microspheres by microfluidics included: 1) two immiscible solvents; 2) devices resistant to both solvents; 3) a solvent with high solubility for PLGA and the drug; 4) a suitable method to remove the dispersed phase solvent; and 5) a suitable surfactant to be dissolved in either of the two solvents. In our previous research, HFE-7500 oil was used as the continuous phase solvent to prepare W/O emulsion by COC microfluidic chips.^53^ Meanwhile, 008 Fluoro Surfactant was the corresponding surfactant soluble in HFE oil for emulsion preparation. To use this combination as the continuous phase, we needed another solvent for the dispersed phase that would be immiscible with HFE, not affect COC yet have high solubility for PLGA and the drug. We found that acetonitrile could meet all of these requirements. The solubility of PLGA and vortioxetine in acetonitrile was more than 100 and 10 mg/mL, respectively. Using a high concentration of PLGA could increase the production efficiency, reduce the extent of shape deformation, and ensure good drug distribution. The high drug solubility could keep the drug ratio in the microspheres at a high level when using a high concentration of PLGA. The latent heat of vaporization of HFE and acetonitrile is stated as 88.5 and 729 kJ/kg, respectively, and their densities are 1614 and 786 kg/m^3^ respectively (manufacturer’s information). Although HFE evaporates faster than acetonitrile, acetonitrile is in the upper layer when mixed with HFE, meaning the acetonitrile could be evaporated before the HFE. The evaporation order of solvents was critical because the droplets needed to be solidified while the emulsion was in the continuous phase.

**Figure 1:**
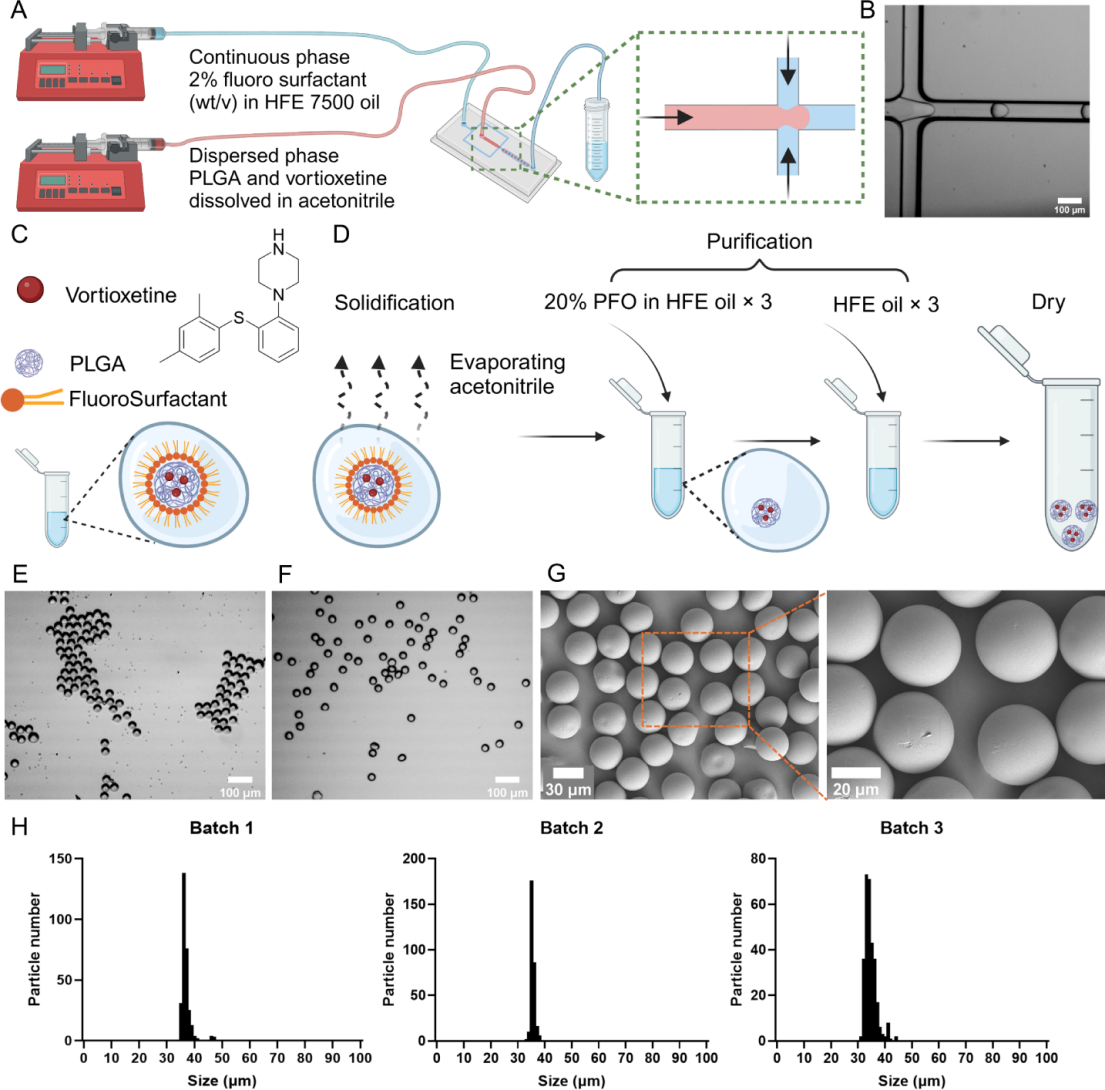
Preparation and characterization of vortioxetine microspheres. (A) A schematic diagram shows the preparation of the O/O (acetonitrile in HFE oil) emulsion by microfluidic techniques. (B) A photo shows droplet generation as a dripping regime at the crossing junction. (C) The structure of the droplets and vortioxetine. (D) A schematic diagram showing the procedure of the purification. Droplets were solidified by evaporating acetonitrile. Fluoro Surfactant was removed by PFO. Dry powder microspheres were produced after the HFE oil was evaporated. Representative bright field microscopy images of (E) droplets and (F) vortioxetine microspheres after purification. (G) SEM pictures and a component of the zoomed-in portion of vortioxetine microspheres. (H) Shows the size distribution of vortioxetine microspheres from three batches (*n* = 300 per batch). Figure (A, C, and D) is generated with Biorender.com.

In order to achieve monodisperse droplets via microfluidics (Figure 1A), a dripping regime was used, as shown in Figure 1B.^54,55^ The high viscosity of the dispersed phase when using high molecular weight PLGA led to unstable droplet generation (Figure S1), so PLGA (L:G 75:25) of molecular weight 4000–15000 Da was used. The schematic diagram (Figure 1C) depicts the structures of the droplets. The perfluoropolyether/poly(ethylene glycol) (PFPE–PEG–PFPE) triblock copolymer surfactant gave remarkable stability to the formed droplets, allowing ease of handling during purification, etc. Furthermore, it was removed easily due to its high solubility in PFO. The procedure for solidification of the droplets and the purification of PLGA microspheres is shown in Figure 1D. Figure 1E, F shows the morphology of the droplets and the purified vortioxetine microspheres that were resuspended in PBS. The size of the vortioxetine microspheres was slightly decreased compared to the droplets due to the evaporation of acetonitrile. SEM images (Figure 1G) show that the microspheres had a regular spherical shape with a smooth surface. Three batches of vortioxetine microspheres were prepared to evaluate reproducibility. All batches of microspheres had a similar size with a narrow size distribution (Batch 1: 36.80 ± 1.96 μm, Batch 2: 35.41 ± 1.05 μm, Batch 3: 34.49 ± 2.15 μm; *n* = 300 for each batch; Figure 1H). Empty PLGA microspheres were also prepared by keeping the concentration of PLGA constant. The size of empty PLGA microspheres was 33.44 ± 1.86 μm (*n* = 300), slightly smaller than the vortioxetine microspheres. The size distribution of empty PLGA microspheres is shown in Figure S2.

Figure S3A shows the FTIR spectra of PLGA, vortioxetine, vortioxetine microspheres, and a physical mixture of vortioxetine and PLGA powder. The peaks of vortioxetine microspheres and the physical mixture did not change in comparison to the PLGA material, indicating no chemical interactions between vortioxetine and PLGA. The proportion of vortioxetine in the physical mixture affected the intensity of the signature peaks of vortioxetine (Figure S3B). The characteristic peaks of vortioxetine, related to N–H stretching at 3240 cm^–1^, =C–H stretching in aromatic at 3050 cm^–1^, and C=C stretching in an aromatic ring at 1580 and 1470 cm^–1^, presented in the 1:1 weight ratio physical mixture without shifts, further confirming no chemical interactions.

### 3.2 High Vortioxetine Loading Efficiency is Achieved in Microspheres

**Figure 2:**
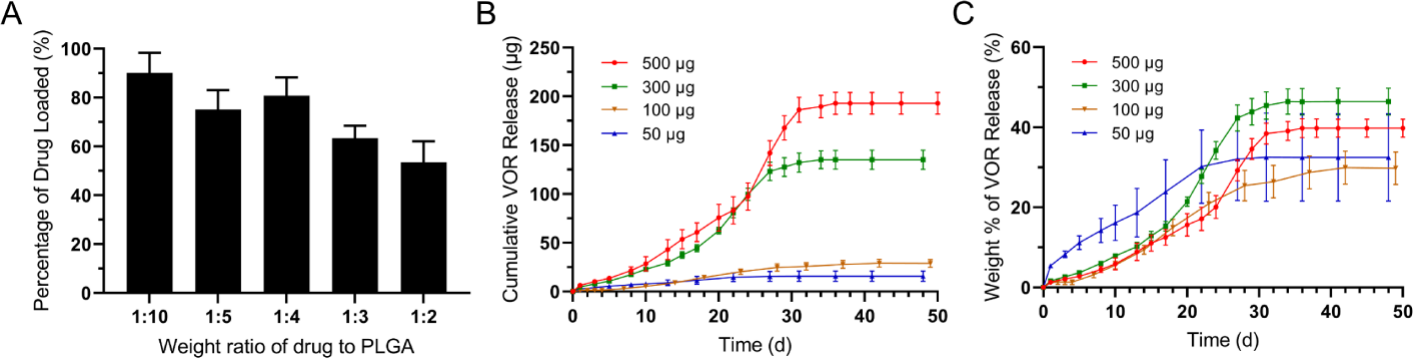
High drug loading efficiency and sustained drug release can be achieved with vortioxetine microspheres. (A) Shows the drug loading efficiency of vortioxetine microspheres at various weight ratios of drug to PLGA (*n* = 3, error bars represent the mean ± SD). (B,C) Show the amount and the weight percentage of cumulative drug release over time from the vortioxetine microspheres (1:10 weight ratio) (*n* = 4, error bars represent the mean ± SD). The legend shows the amount of vortioxetine in the microspheres before release. Abbreviation: VOR: vortioxetine.

To investigate the drug-loading efficiency, vortioxetine microspheres with different weight ratios of drug to PLGA were prepared. As shown in Figure 2A, a 1:10 drug-to-polymer ratio showed the highest loading efficiency, with 90.06% ± 8.27% (*n* = 3) of the drug being entrapped in the microspheres. As expected, the loading efficiency decreased when the ratio of drug to PLGA increased, as drug molecules might leak from the droplets during solidification due to the reduced PLGA concentration. Therefore, a 1:10 ratio was used for the subsequent experiments (percentage of drug loading: 9.1%(w/w)).

### 3.3 Sustained Vortioxetine Release from the Microspheres

The drug release study was conducted in a pure PBS medium. As shown in Figure 2B, C, vortioxetine microspheres did not show a burst release and could sustain drug release for more than one month. Samples with 300 and 500 μg of drug had an obvious biphasic drug release pattern, while 50 and 100 μg samples did not show a significant increase in release rate during the experiment. The drug release rate was nearly zero-order kinetics for the first phase. The release rate of the 300 and 500 μg samples increased between days 27–31 and days 20–27, respectively. Large microspheres usually have a triphasic release profile.^56^ The drug trapped on or close to the surface of the microspheres is released in the initial burst release.^57^ PLGA microspheres with high porosity at the surface have previously exhibited a burst release pattern.^58^ The sigmoidal shape of the drug release pattern was likely due to the degradation and erosion of PLGA, creating pores from which encapsulated drugs are released by diffusion. The absence of a burst release in these studies may be due to an even drug distribution in the polymer network and a smooth/low-porosity surface for degradation/erosion to occur.

### 3.4 Cytocompatibility of Empty Microspheres

**Figure 3:**
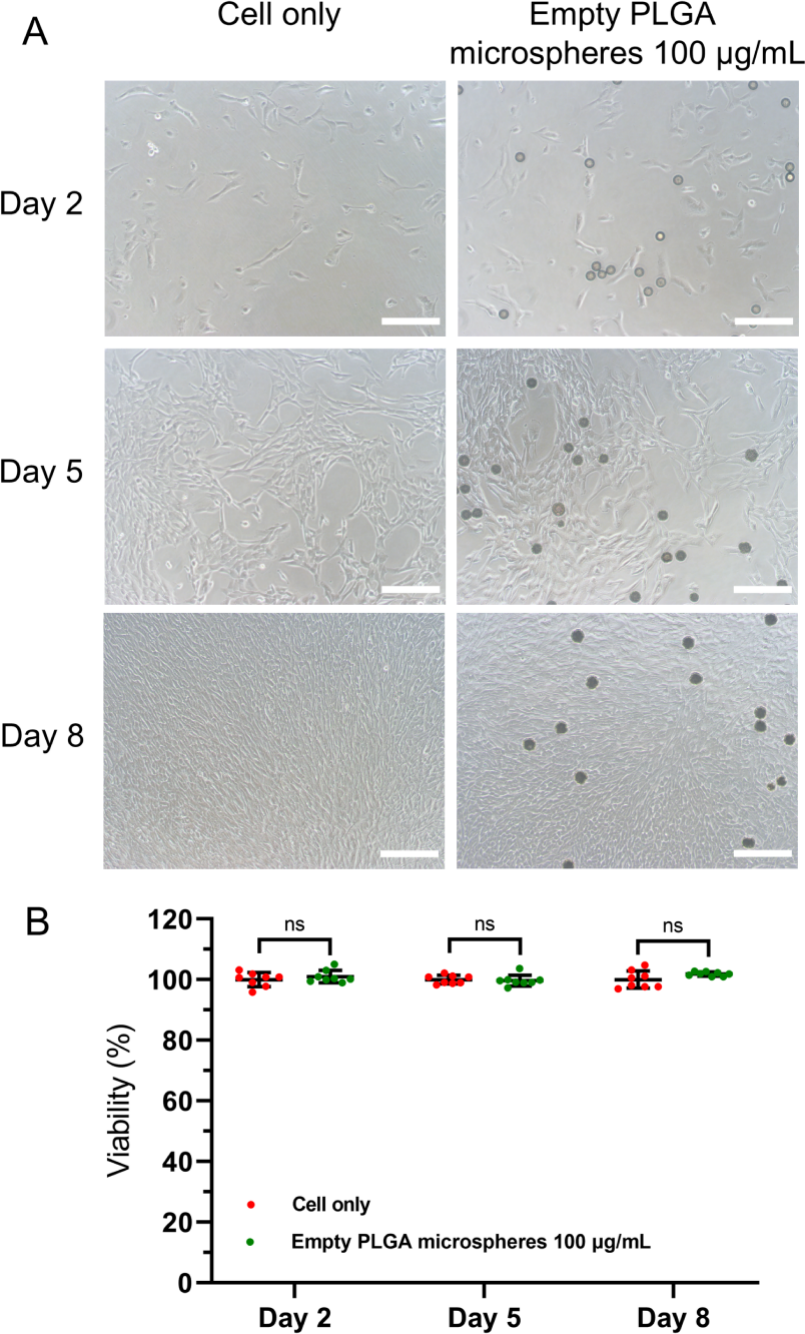
Empty PLGA microspheres cause no toxicity to primary human astrocytes. (A) Shows representative images of human astrocytes incubated with or without 100 μg/mL empty PLGA microspheres. Empty PLGA microspheres were added on day 1. The dark circular shapes are the microspheres. Images were taken on days 2, 5, and 8 (scale bar = 200 μm). (B) Shows the cell viability on days 2, 5, and 8 (*n* = 8, error bars represent the mean ± SD). Unpaired *t*-test for day 2 and day 5, Welch’s *t*-test for day 8, ns = no significant difference.

Astrocytes were used to evaluate the cytocompatibility of empty PLGA microspheres due to their high prevalence and key support role in the central nervous system. 100 μg/mL empty PLGA microspheres were incubated with human astrocytes (hAstrocytes) to match the highest concentration of vortioxetine microspheres used in subsequent experiments. As shown in Figure 3A, no changes in the morphology of hAstrocytes were observed when incubated with the microspheres, nor was any reduction in viability at days 1, 4, or 7 (Figure 3B). These results indicate that the new preparation method for PLGA microspheres does not incorporate any toxic compounds into the final product.

### 3.5 Cytotoxicity of Free Vortioxetine

The cytotoxicity of free vortioxetine was investigated on hAstrocytes and three patient-derived GBM cells (hGBM L0, L1, and L2). The differences in protein expression among these three GBM cell lines included only hGBM L1 expressing OLIG2 and CD44, and hGBM L2 not expressing TOP2A and NF1.^44^ The cytotoxicity of the solvent solution (DMSO in PBS) was checked, and the results showed that cell viability was not affected (Figure S4). As shown in Figure S5, vortioxetine exhibited time-dependent and dose-dependent toxicity. The IC_50_ values for all hGBM cell lines were around 1 μg/mL on day 1 (Table 1). On day 5, hGBM L2 was the most sensitive hGBM cell line to vortioxetine, while hGBM L0 was the most resistant cell line. However, the IC_50_ value of hAstrocytes was doubled, even compared to hGBM L0 (1.48 μg/mL vs 0.75 μg/mL), which meant that there was a potentially decent therapeutic window to minimize side effects on healthy/nonmalignant cells. The IC_50_ values of free Temozolomide, the first-line chemotherapeutic agent in clinical use for glioblastoma, on hAstrocytes and hGBM cell lines were tens of times higher than those of vortioxetine (e.g., 311.9 μg/mL on hAstrocytes and 23.09 μg/mL on hGBM L0 treated for 4 days; Figure S6, Table S1). Although Temozolomide also had a therapeutic window, it was more toxic toward hAstrocytes on day 8 compared to day 5, indicating delayed but substantial toxicity toward hAstrocytes.

**Table 1:** IC_50_ Values of Free Vortioxetine on hGBM Cells and Human Astrocytes (μg/mL)

	hGBM L0	hGBM L1	hGBM L2	hAstrocytes
Day 2	1.02	1.06	1.25	2.41
Day 5	0.75	0.62	0.39	1.48
Day 8	0.77	0.65	0.47	1.44

Our previous research showed that the IC_50_ value for the LN-229 GBM cell line was around 5 μM (1.5 μg/mL) and around 20 μM (6.0 μg/mL) for the ZH-161 cell line after 48 h treatment.^17^ The *in vivo* dosage for these two types of tumor models was 10 mg/kg via intraperitoneal administration, which showed significant survival benefits compared to the negative control. Based on the IC_50_ values for hGBM cell lines, the growth of these tumor models in an animal study should be effectively suppressed by vortioxetine.

### 3.6 Anti-GBM Efficacy of Vortioxetine Microspheres

**Figure 4:**
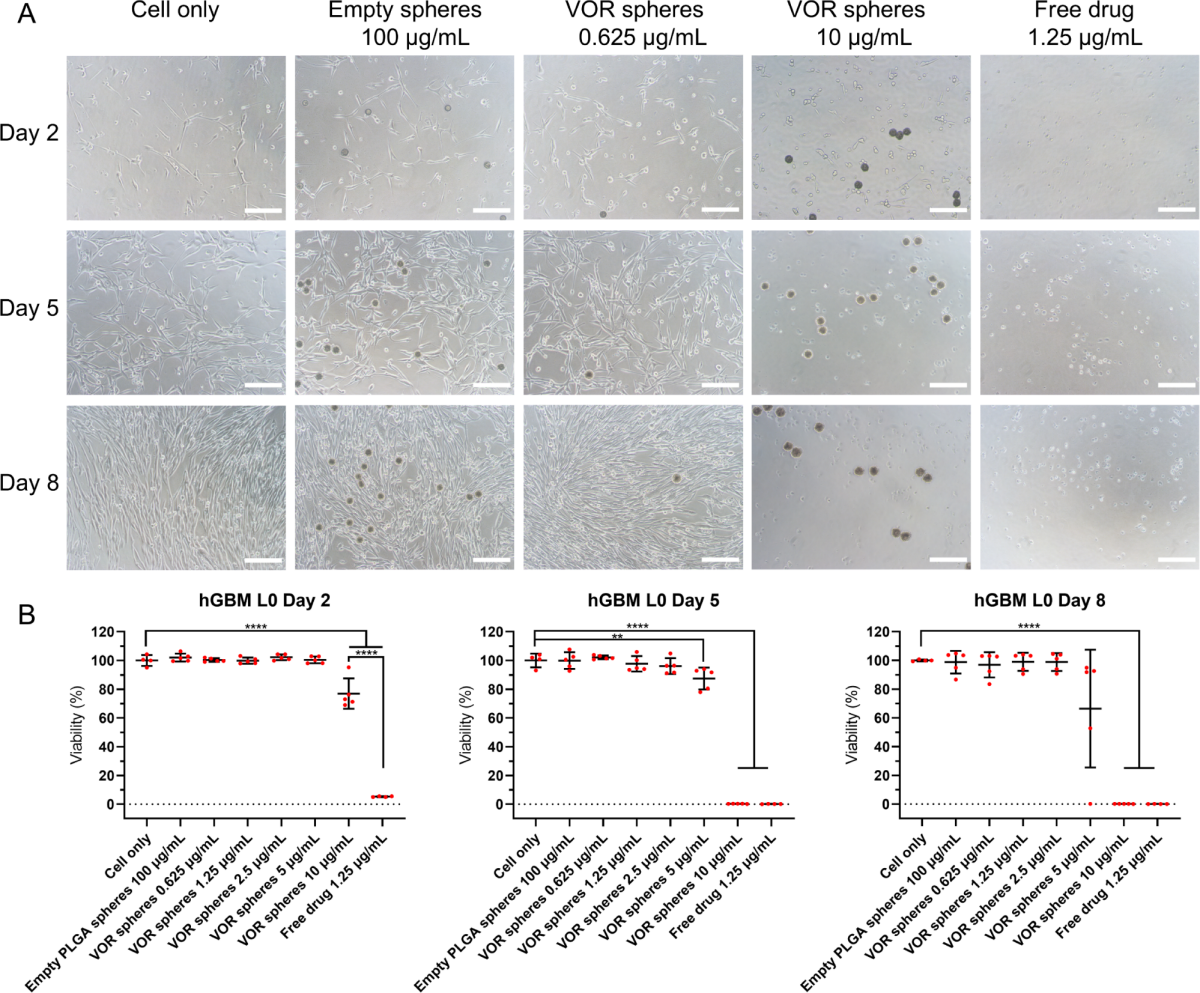
Vortioxetine microspheres reduce the viability of hGBM L0 cells. (A) Shows representative bright field images of hGBM L0 cells incubated with culture medium only, empty PLGA microspheres 100 μg/mL, vortioxetine microspheres 10 or 0.625 μg/mL, and free vortioxetine 1.25 μg/mL. PLGA microspheres were added on day 1. Images were taken on days 2, 5, and 8 (scale bar = 200 μm). The dark circular shapes are the microspheres. (B) Shows the cell viability on days 2, 5, and 8 (empty PLGA microspheres and vortioxetine microspheres, *n* = 5; cell only and free drug, *n* = 4; mean ± SD). Ordinary one-way ANOVA test for all time points, for **p ≤ 0.01, and ****p ≤ 0.001. Abbreviation: VOR: vortioxetine.

**Figure 5:**
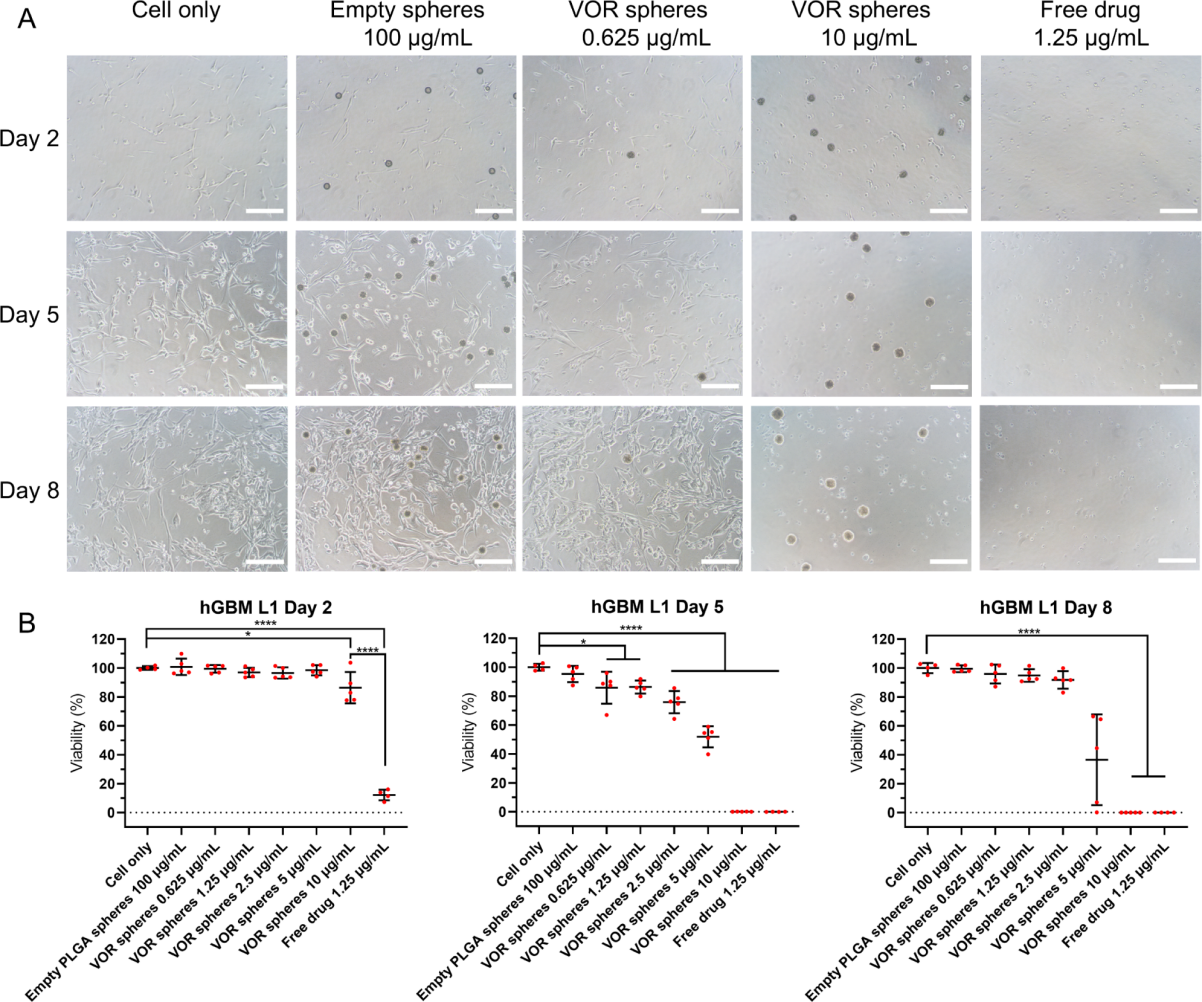
Vortioxetine microspheres reduced the viability of hGBM L1 cells. (A) Shows representative bright field images of hGBM L1 cells incubated with culture medium only, empty PLGA microspheres 100 μg/mL, vortioxetine microspheres 10 or 0.625 μg/mL, and free vortioxetine 1.25 μg/mL. PLGA microspheres were added on day 1. Images were taken on days 2, 5, and 8 (scale bar = 200 μm). The dark circular shapes are the microspheres. (B) Shows the cell viability on days 2, 5, and 8 (empty PLGA microspheres and vortioxetine microspheres, *n* = 5; cell only and free drug, *n* = 4; mean ± SD). Ordinary one-way ANOVA test: day 2 and day 5, Welch ANOVA test: day 8, for *p ≤ 0.05, ****p ≤ 0.001. Abbreviation: VOR: vortioxetine.

**Figure 6:**
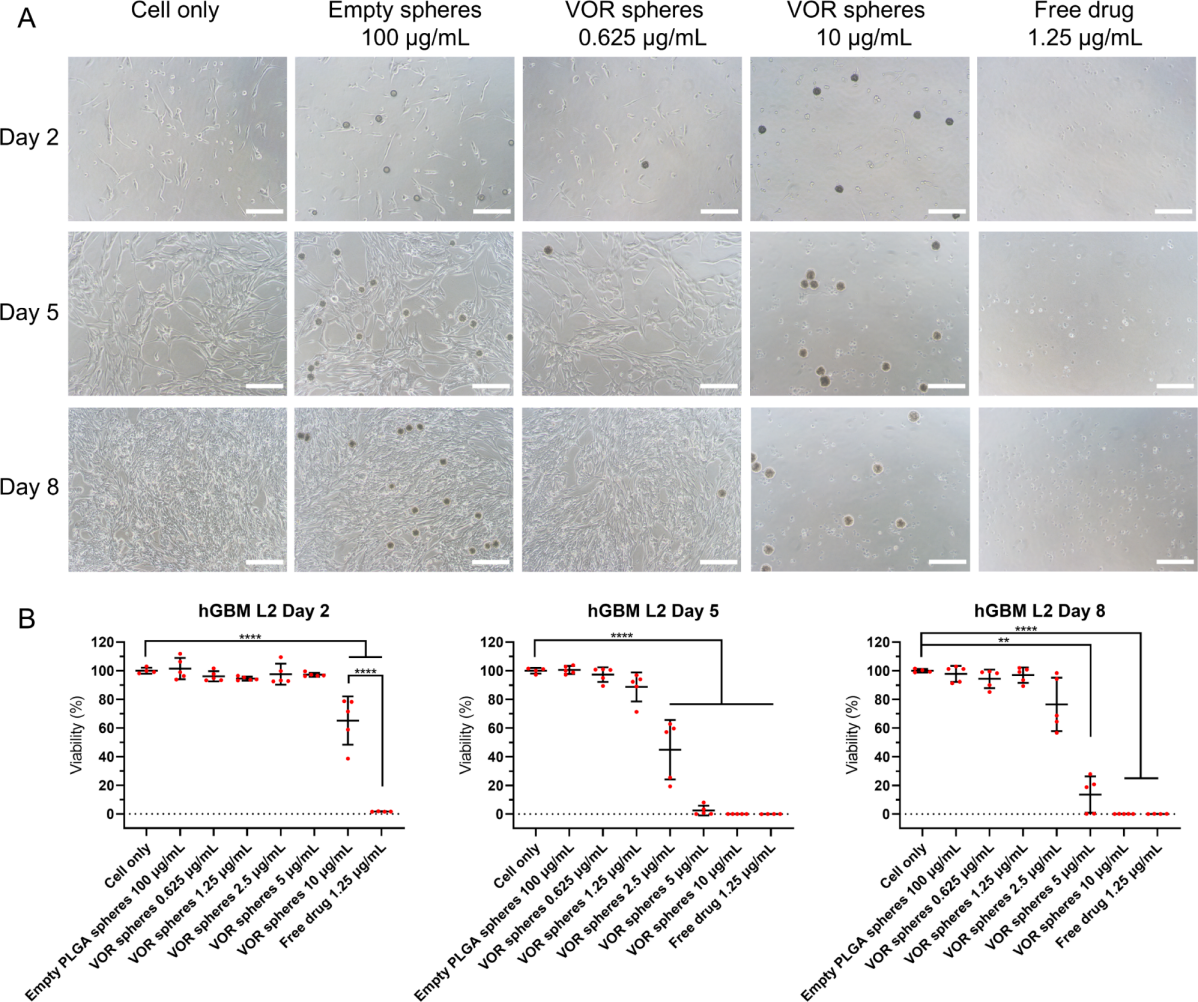
Vortioxetine microspheres reduce the viability of hGBM L2. (A) Shows representative bright field images of hGBM L2 cells incubated with culture medium only, empty PLGA microspheres of 100 μg/mL, vortioxetine microspheres of 10 or 0.625 μg/mL, and free vortioxetine of 1.25 μg/mL. PLGA microspheres were added on day 1. Images were taken on days 2, 5, and 8 (scale bar = 200 μm). The dark circular shapes are the microspheres. (B) Shows the cell viability on days 2, 5, and 8 (empty PLGA microspheres and vortioxetine microspheres, *n* = 5; cell only and free drug, *n* = 4; mean ± SD). Ordinary one-way ANOVA test: day 2 and day 5, Welch ANOVA test: day 8, for **p ≤ 0.01, ****p ≤ 0.001. Abbreviation: VOR: vortioxetine.

Geltrex basement membrane matrix was used to culture hGBM cell lines as adherent cells to investigate the *in vitro* antitumor efficacy of vortioxetine microspheres in 2D models. As shown in Figures 4–6, empty PLGA microspheres did not reduce the viability of hGBM cells after 7 days of incubation (hGBM L0: 98.78% ± 7.86%; hGBM L1: 99.51% ± 2.38%; hGBM L2: 97.80% ± 5.55%; *n* = 5 for each cell line). 1.25 μg/mL free drug killed nearly all tumor cells after 1 day of incubation (hGBM L0: 5.28% ± 0.36%; hGBM L1: 12.15% ± 3.73%; hGBM L2 :1.68% ± 0.17%; *n* = 4 for each cell line). Vortioxetine microspheres showed time- and dose-dependent efficacy. Compared with cells treated with 1.25 μg/mL free drug, the cell viability of 10 μg/mL vortioxetine microspheres on day 2 (hGBM L0:76.97% ± 10.54%; hGBM L1 :86.34% ± 10.08%; hGBM L2: 65.17% ± 16.89%; *n* = 5 for each cell line) was significantly higher, which confirmed that the microspheres did not exhibit a burst release of the drug. The sensitivity of the three hGBM cell lines to vortioxetine also matched the previous IC_50_ experiment (Figure S5). This can be concluded because the drug release data showed about 6%, 15%, and 20 payload release after 1, 4, and 7 days of incubation, respectively, which fits with the microsphere efficacy data (Figures 4–6) and with the dose–response curve of free vortioxetine (Figure S5). The cytotoxicity of vortioxetine microspheres was also evaluated on hAstrocytes (Figure S7). The cell viability was 58.63% ± 11.53% (*n* = 5) after being treated with 10 μg/mL vortioxetine microspheres for 7 days, which was much higher than that of hGBM cell lines. Five μg/mL vortioxetine microspheres did not significantly reduce the cell viability of hAstrocytes (87.50% ± 7.51%, *n* = 5) compared to the negative control (*p* = 0.1831) on day 8, but this concentration was not enough to kill resistant tumor cells such as hGBM L0. So, 5–10 μg/mL is the optimal dose range for vortioxetine microspheres to eradicate GBM cells, depending on the resistance of the cell lines.

### 3.7 Vortioxetine Microspheres Effectively Destroy a hGBM 3D Tumor Spheroid Model

Monolayer cell cultures lack physiological relevance to the *in vivo* environment, perhaps contributing to the high failure rate of drug candidates entering phase I trials.^59^ 3D cell culture models have gained more attraction in drug discovery because they better mimic the *in vivo* situation in terms of cell proliferation, cell–cell interaction, and protein expression.^60^ 3D-cultured hepatocellular carcinoma cells showed organoid-like features that mimicked the *in vivo* conditions of glandular epithelium, such as acinar morphogenesis and the expression of progenitor cell markers.^61^ Oskarsson et al. found that 3D tumor spheroids upregulated the expression of embryonic stem cell markers while they downregulated the expression of differentiation markers compared to monolayer cell culture, which suggested a correlation to the *in vivo* microenvironment in stem cell niches.^62^ 3D models have also exhibited increased expression of drug resistance-related genes,^63^ showing mimicry of the in vivo condition. Furthermore, 3D cell culture models showed an enhancement of GBM stemness and chemotherapy resistance compared to 2D cell culture models.^64^ Ma et al. found that the gene expression of GBM cells dramatically changed when comparing 2D and 3D cell culture models^65^ and indicated that a 3D cell culture model was a more relevant platform for drug screening. We used round-bottom ultralow attachment well plates to culture hGBM L2 as 3D spheroids via the forced-floating method.

**Figure 7:**
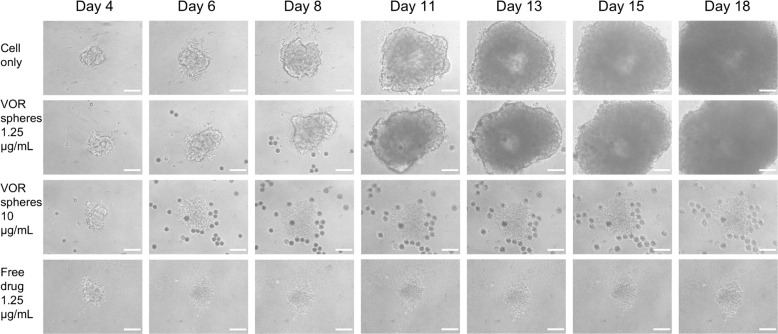
Vortioxetine microspheres suppress the growth of the hGBM L2 spheroids. Representative bright field images of hGBM L2 3D spheroids incubated with culture medium only, vortioxetine microspheres 10 or 1.25 μg/mL, and free vortioxetine 1.25 μg/mL (scale bar = 200 μm). PLGA microspheres were added on day 4 and can be seen as dark circular shapes. Abbreviation: VOR: vortioxetine.

**Figure 8:**
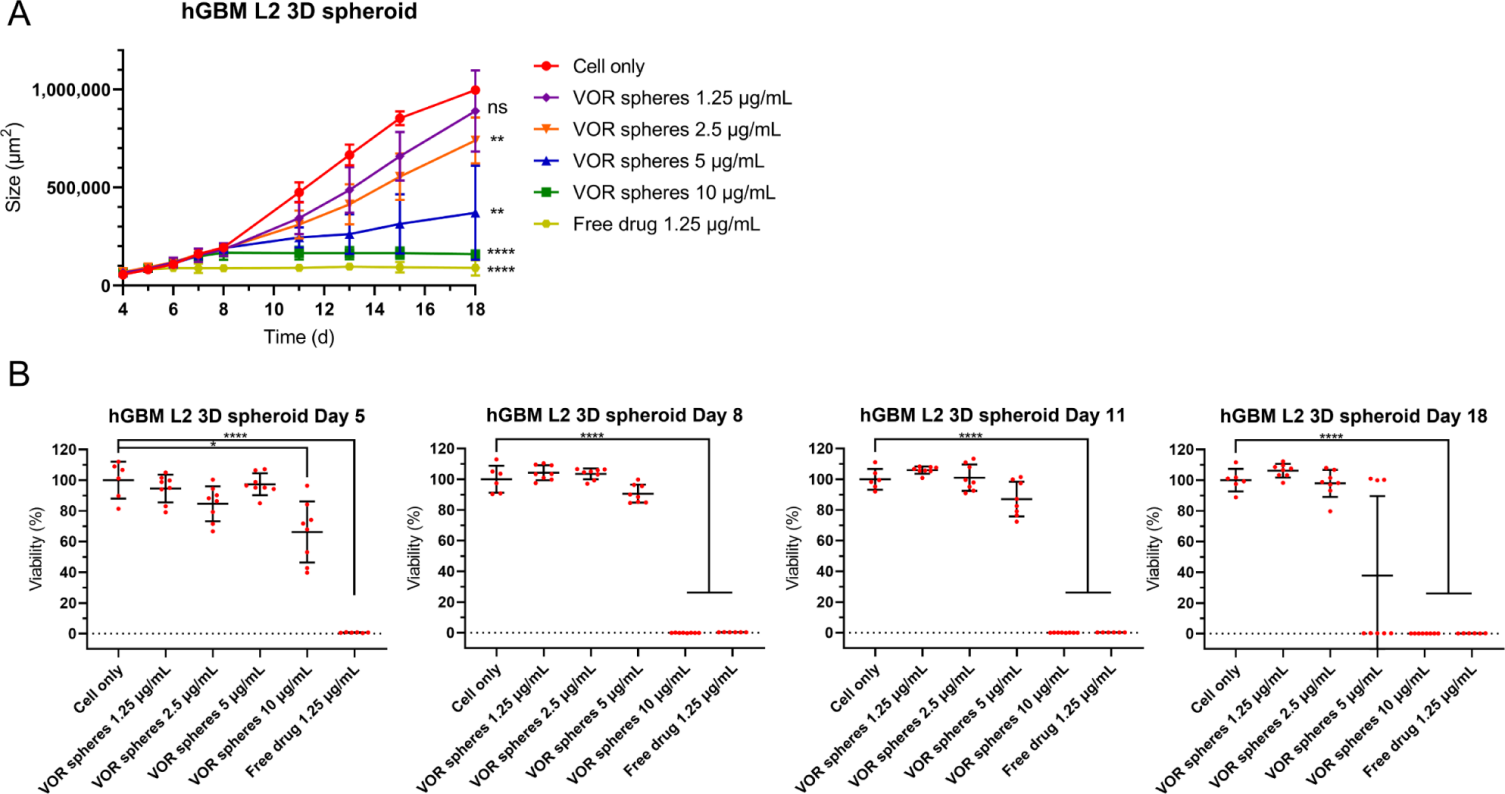
Vortioxetine microspheres reduce the cell viability of hGBM L2 spheroids. (A) Shows the size of hGBM L2 3D spheroids incubated with vortioxetine microspheres (cell only and free drug, *n* = 6; vortioxetine microspheres, *n* = 8; mean ± SD). (B) Shows the cell viability on days 5, 8, 11, and 18 (cell only and free drug, *n* = 6; vortioxetine microspheres, *n* = 8; mean ± SD). (A) Two-way ANOVA test. The statistically significant difference compared to cell only. (B) Welch ANOVA test. For ns = no significant difference, *p ≤ 0.05, **p ≤ 0.01, ****p ≤ 0.001. Abbreviation: VOR: vortioxetine.

Figure 7 shows representative images of hGBM L2 spheroids cultured with 1.25 or 10 μg/mL vortioxetine microspheres, 1.25 μg/mL free drug, or without treatment. The alive spheroids had a clear edge and became less transparent during the experiment. Dead spheroids lost cell attachments and broke off, resulting in a larger area of dead cells than the spheroid. Comparing the images on day 4 and day 6, the size of the PLGA microspheres grew through swelling in the aqueous medium (the dark circles scattered throughout the image are the microspheres). The PLGA microspheres kept their integrated structure even after 14 days of incubation. Compared to the cell-only control, vortioxetine microspheres suppressed the growth of cell spheroids in a dose-dependent manner (Figure 8A). The size of cell spheroids on day 18: cell-only control: 997,243 ± 20,954 μm^2^, *n* = 6; 1.25 μg/mL vortioxetine microspheres: 890,154 ± 207,720 μm^2^, *n* = 8; 2.5 μg/mL vortioxetine microspheres: 740,423 ± 108,214 μm^2^, *n* = 8; 5 μg/mL vortioxetine microspheres: 371,084 ± 245,057 μm^2^, *n* = 8; 10 μg/mL vortioxetine microspheres: 106,660 ± 12,100 μm^2^, *n* = 8; 1.25 μg/mL free drug: 90,434 ± 38,988 μm^2^, *n* = 6. 1.25 μg/mL vortioxetine microspheres could not reduce the growth of cell spheroids during the 14-day period. However, when the concentration reached 2.5 μg/mL, cell spheroids were significantly smaller than those in the no-treatment group. 5 μg/mL vortioxetine microspheres killed 5 of 8 cell spheroids during the 14 days (Figure 8B). Ten μg/mL vortioxetine microspheres resulted in the complete destruction of all the spheroids after 4 days. Directly adding 1.25 μg/mL free vortioxetine resulted in the rapid killing of the spheroids after only 1 day (cell viability: 0.73% ± 0.21%, *n* = 6). Together, these results confirm that vortioxetine microspheres exhibited no initial burst release, which could have compromised their safety.

Although the cell viability of cell spheroids cultured with 2.5 and 5 μg/mL vortioxetine microspheres was not significantly different from the nontreatment group, these concentrations could effectively suppress the growth of hGBM cell spheroids. Five μg/mL vortioxetine microspheres significantly decreased the cell viability of the hGBM L2 2D culture model by day 8, but not in the 3D culture model, indicating higher drug resistance in the 3D culture model. This could potentially be due to differences in cell proliferation, metabolism, and communication between 2D and 3D cultures. In the most resistant hGBM L0 model, 10 μg/mL vortioxetine microspheres killed the spheroids in the early stage of spheroid development, but 5 μg/mL vortioxetine microspheres did not suppress cell growth (Figures S8 and S9). Next, the efficacy of vortioxetine microspheres against a grown 3D spheroid was evaluated by postponing the starting date of the treatment to day 7. Ten μg/mL vortioxetine microspheres significantly decreased the size of the hGBM L0 spheroids compared to the negative control on day 16 (932,034 ± 151,200 μm^2^ vs 1,269,770 ± 71,657 μm^2^, *n* = 8, Figures S10 and S11). The no significant differences in cell viability between the vortioxetine microsphere treatment and the control group on day 16 (Figure S11C) might be because of the limited penetration of the 3D spheroids for the PrestoBlue reagent. Similar results were observed for hGBM L2 spheroids (Figures S12 and S13). These results further proved the efficacy of vortioxetine against GBM in all experimental parameters tested, providing a rationale for further analysis as a new therapeutic strategy for glioblastoma.

## 4 Conclusions

In summary, we developed a new method to prepare PLGA microspheres by droplet-based microfluidics with an oil-in-oil emulsion formula. Vortioxetine, the drug proposed to be repurposed as a GBM therapy, was loaded into the PLGA microspheres with high loading efficiency. Drug release patterns showed that PLGA microspheres released the drug in a biphasic manner, and no initial burst release was observed. Drugs were released with near zero-order kinetics for around 3 weeks, followed by 1 week of faster, degradation-controlled release. Empty PLGA microspheres exhibited good cytocompatibility toward astrocytes. The IC_50_ value of free vortioxetine on astrocytes vs glioblastoma cells suggested that there is a therapeutic window, minimizing toxicity to healthy cells. Vortioxetine microspheres showed efficacy against hGBM cells in both 2D monolayer cell culture models and 3D spheroid cell culture models. The optimal dose range for vortioxetine microspheres is 5–10 μg/mL, depending on the therapeutic resistance of the GBM cells. These results have shown that vortioxetine could have therapeutic potential for GBM and can be delivered in a controlled and sustained manner from microspheres.

## Supporting Information Available

Images show the droplet generation regime; the size distribution of empty PLGA microspheres; FTIR spectra of vortioxetine microspheres, a physical mixture of PLGA and vortioxetine, free drug, and PLGA materials; dose-respond curves of free vortioxetine and Temozolomide on three patient-derived GBM cell lines and human astrocytes; IC_50_ values of free Temozolomide on all four cell lines; the DMSO concentration used does not affect cell viability; the cytotoxicity of vortioxetine microspheres on human astrocytes; representative bright field images of hGBM L0 3D spheroids during treatment with vortioxetine loaded spheres and free drug; vortioxetine microspheres eradicate the more resistant hGBM L0 3D spheroids at the higher concentration; representative bright field images show vortioxetine microspheres against the pre-established (grown) hGBM L0 3D spheroids; vortioxetine microspheres reduce the size of the pre-established (grown) hGBM L0 3D spheroids; representative bright field images show vortioxetine microspheres against the pre-established (grown) hGBM L2 3D spheroids; vortioxetine microspheres reduce the size of the pre-established (grown) hGBM L2 3D spheroids (PDF)
