# Supplementary material for: Vortioxetine: A Potential Drug for Repurposing for Glioblastoma Treatment via a Microsphere Local Delivery System
Source: ACS Biomater Sci Eng. 2025 Apr 1;11(4):2203–15. doi: 10.1021/acsbiomaterials.5c00068 (PMC12001186; doi:10.1021/acsbiomaterials.5c00068)
Supplement: Supplementary file 1 — ab5c00068_si_001.pdf [file ab5c00068_si_001.pdf]

Vortioxetine: a potential drug for repurposing for glioblastoma treatment via a microsphere local delivery system

Yu Wang[a], Dorit Siebzehnrubl[b], Michael Weller[c], Tobias Weiss[c], Florian A. Siebzehnrubl[b], Ben Newland[a]*

[a] School of Pharmacy and Pharmaceutical Sciences, Cardiff University, King Edward VII Avenue, Cardiff, CF10 3NB, United Kingdom

[b] Cardiff University School of Biosciences, European Cancer Stem Cell Research Institute, Cardiff, CF24 4HQ, United Kingdom

[c] Department of Neurology, Clinical Neuroscience Center, University Hospital Zurich and University of Zurich, Zurich, Switzerland

* Corresponding author, electronic address: newlandb@cardiff.ac.uk

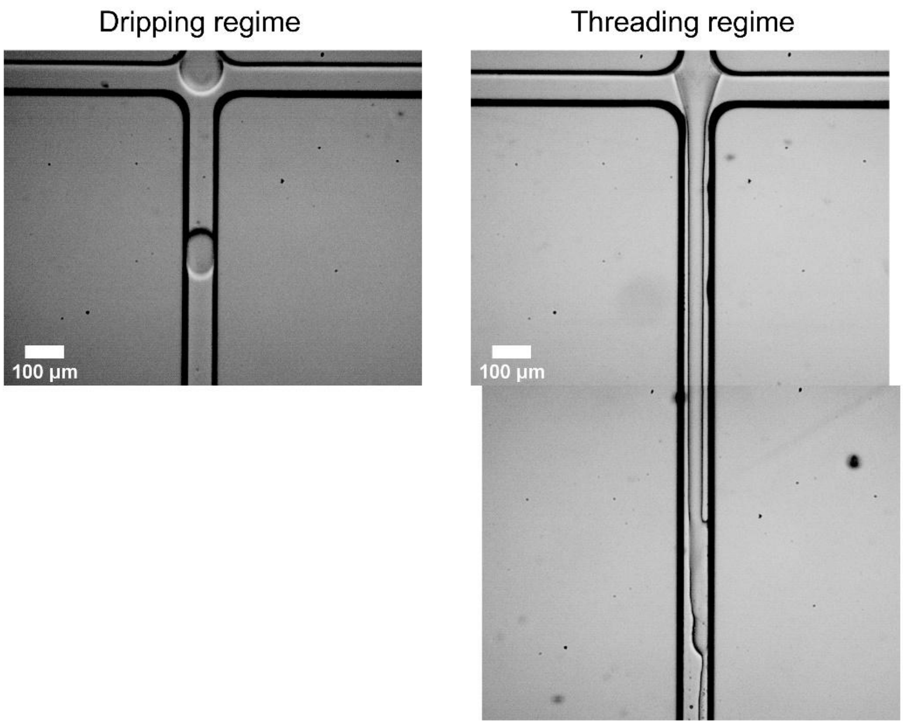

Figure S1. The droplet generation was unstable when using higher molecular weight PLGA (molecular weight range: 76,000-115,000) at a concentration of 100 μg/mL. The dripping regime would switch to the threading regime.

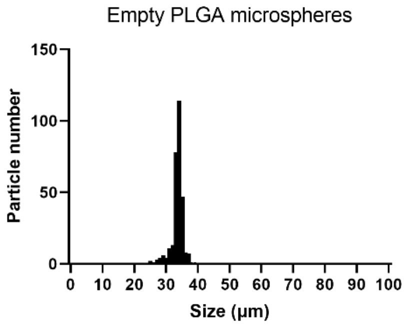

Figure S2. The size distribution of empty PLGA microspheres (n=300).

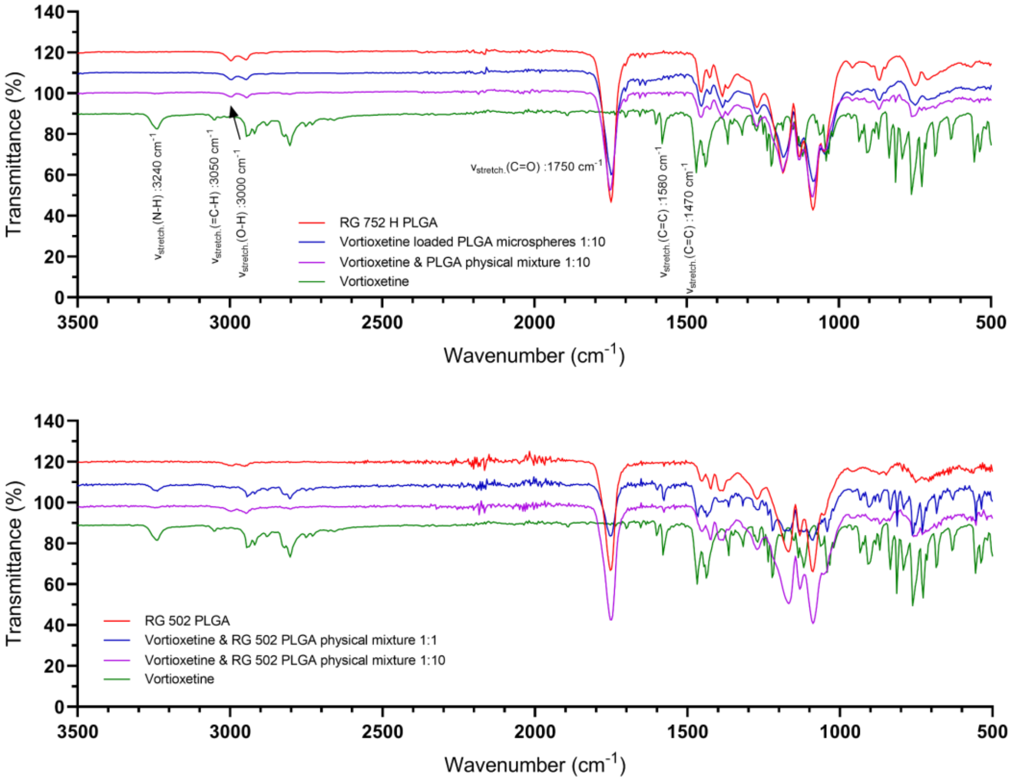

Figure S3. FTIR spectra of vortioxetine microspheres, a physical mixture of PLGA and vortioxetine, PLGA materials, and drug powder. **(A)** shows the FTIR spectra of vortioxetine microspheres and a physical mixture of PLGA and vortioxetine powder with the same weight ratio. **(B)** shows the effect of the vortioxetine proportion in the physical mixture on the intensity of peaks, showing a large reduction in the peaks when only a small amount of drug is present.

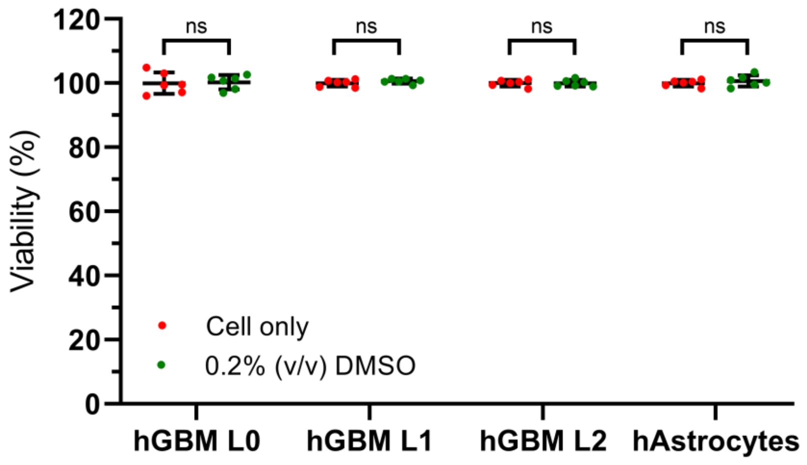

Figure S4. The DMSO concentration used does not affect cell viability. 0.2% (v/v) DMSO in PBS equaled the volume proportion at 10 μg/mL free vortioxetine diluted from the stock solution (5 mg/mL in pure DMSO). At this concentration, no significant reduction in the viability of the three glioblastoma cell types and primary human astrocytes was observed (n=6, error bars represent the mean ± SD; unpaired t-test, ns = no significant difference).

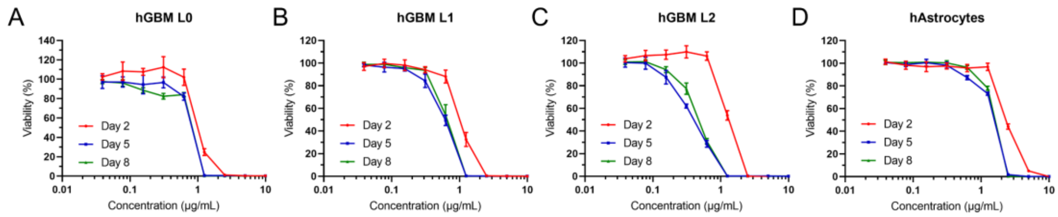

Figure S5. The dose-respond curves of free vortioxetine on hGBM cell lines **(A, B, and C)** and hAstrocytes **(D)** (n=6, error bars represent the mean ± SD).

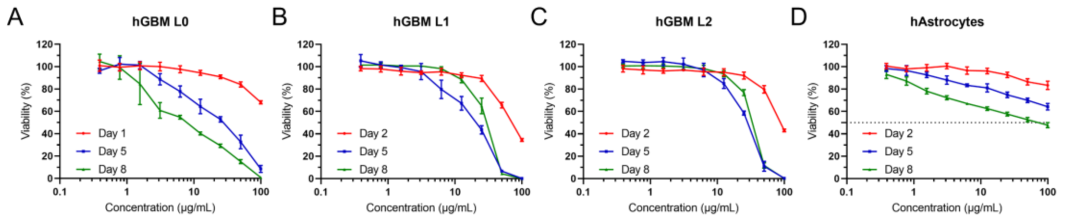

Figure S6. The dose-respond curves of free temozolomide on hGBM cell lines **(A, B and C)** and hAstrocytes **(D)** (n=6, error bars represent the mean ± SD).

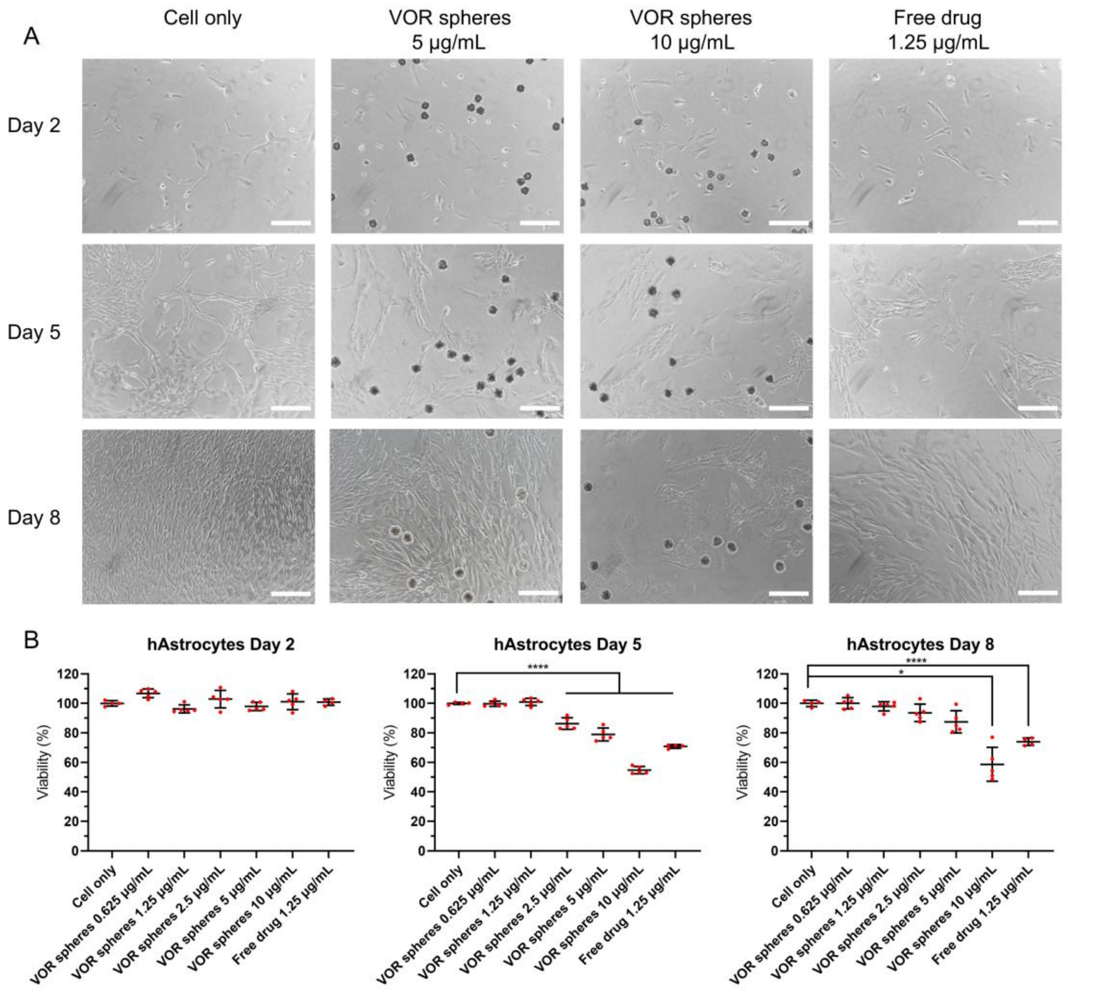

Figure S7. The cytotoxicity of vortioxetine microspheres on primary human astrocytes. **(A)** shows representative bright field images of primary human astrocytes during the treatment. **(B)** shows the cell viability on Day 2, Day 5 and Day 8 (Vortioxetine microspheres, n=5; cell only and free drug, n=4; error bars represent the mean ± SD. Ordinary one-way ANOVA test: Day 2 and Day5, Welch ANOVA test: Day 7, for * $p \leq 0.05$, **** $p \leq 0.001$). PLGA microspheres were added on Day 1. Abbreviation: VOR: vortioxetine.

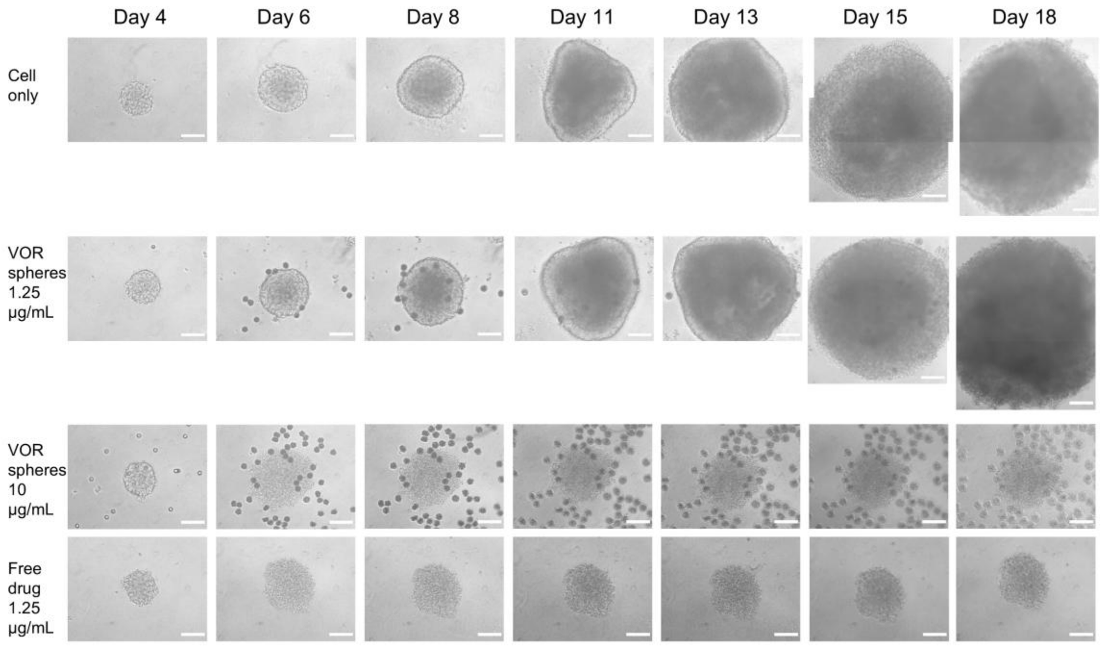

Figure S8. Representative bright field images of hGBM L0 3D spheroids during treatment with vortioxetine loaded spheres and free drug (Scale bar = 200 µm). PLGA microspheres were added on Day 4, and the high concentration resulted in a reduction in spheroid growth. Abbreviation: VOR: vortioxetine.

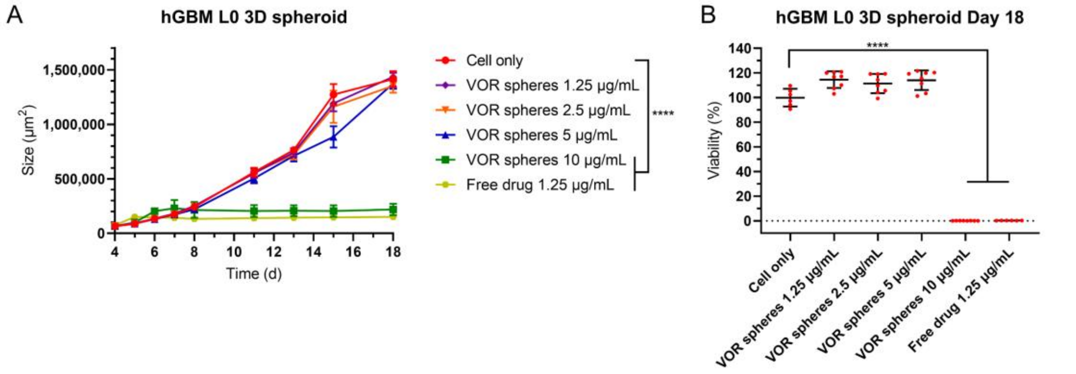

Figure S9. Vortioxetine microspheres eradicate the more resistant hGBM L0 3D spheroids at the higher concentration. **(A)** shows the size of the hGBM L0 3D spheroids during the treatment (Cell only and free drug, n=6; vortioxetine microspheres, n=8. Asterisks represent the statistically significant difference compared to cell only control on Day 18 (Two-way ANOVA test, for **** $p \leq 0.0001$). **(B)** shows the corresponding cell viability at the end of the experiment (Welch ANOVA test). Abbreviation: VOR: vortioxetine. Error bars represent the mean ± SD.

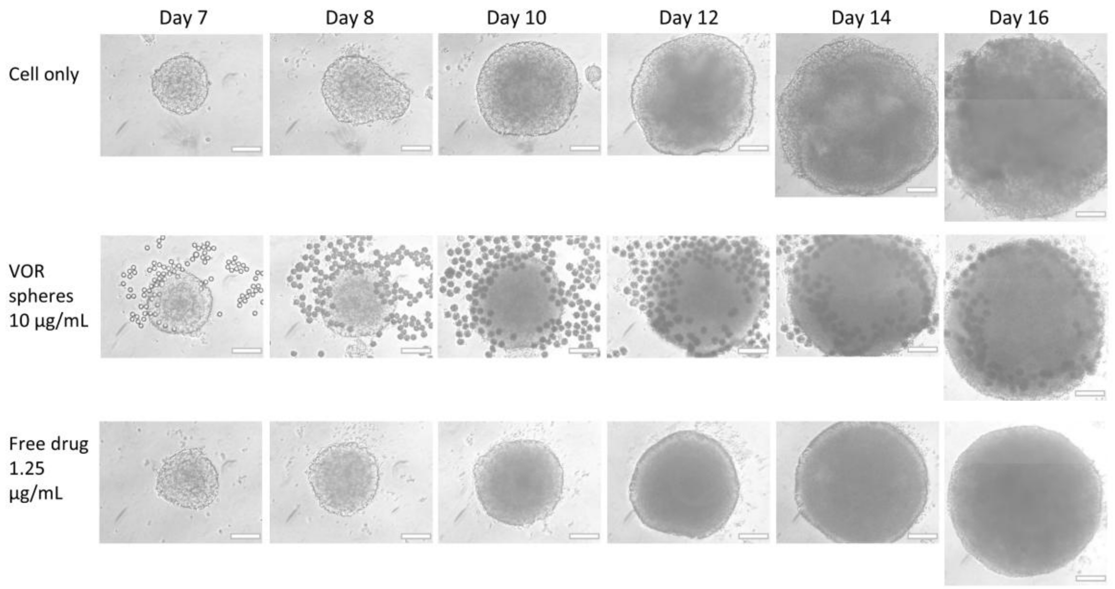

Figure S10. Representative bright field images show vortioxetine microspheres against the pre-established (grown) hGBM L0 3D spheroids (Scale bar = 200 µm). PLGA microspheres were added on Day 7. Abbreviation: VOR: vortioxetine.

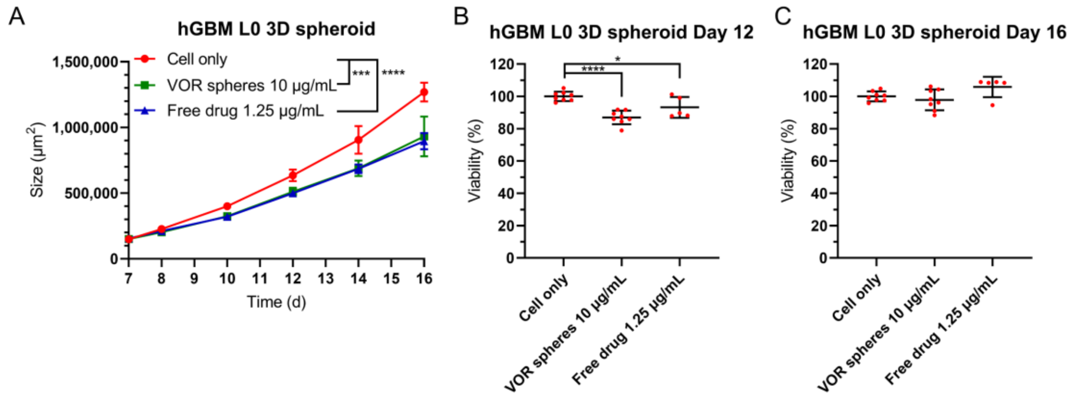

Figure S11. Vortioxetine microspheres reduce the size of the pre-established (grown) hGBM L0 3D spheroids. **(A)** shows the size changes during the treatment. Asterisks represent the statistically significant on Day 16 (Two-way ANOVA test, cell only and vortioxetine microspheres: n=8; free drug: n=5. **(B)** and **(C)** show the cell viability on Day 12 and Day 16, respectively. **(B)**: Ordinary one-way ANOVA test. **(C)**: Nonparametric Kruskal-Wallis test. For * $p \leq 0.05$, *** $p \leq 0.001$, **** $p \leq 0.0001$. PLGA microspheres were added on Day 7. Abbreviation: VOR: vortioxetine. Error bars represent the mean ± SD.

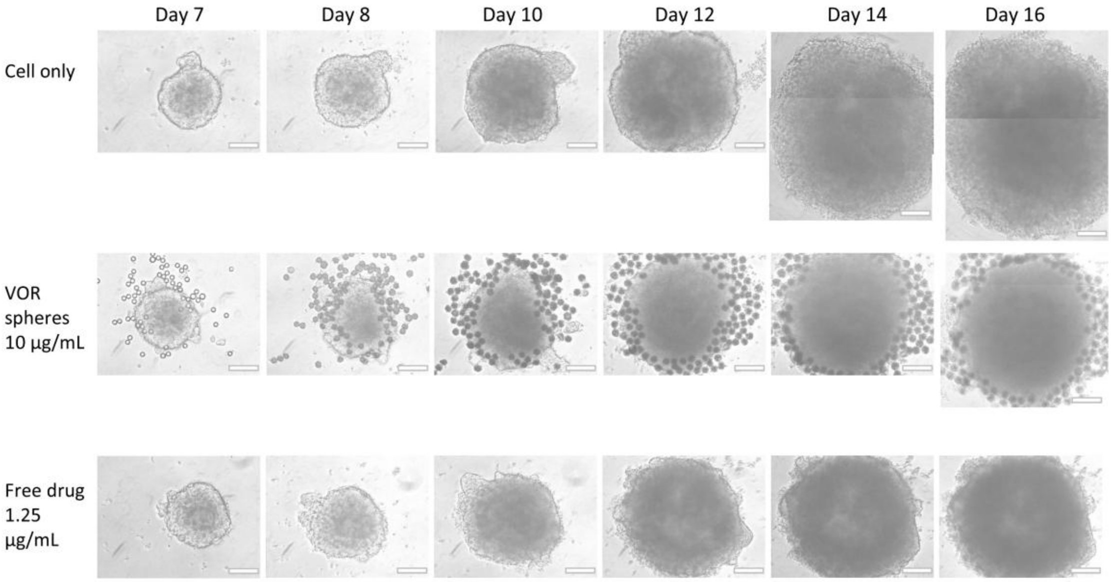

Figure S12. Representative bright field images show vortioxetine microspheres against the pre-established (grown) hGBM L2 3D spheroids (Scale bar = 200 µm). PLGA microspheres were added on Day 7. Abbreviation: VOR: vortioxetine.

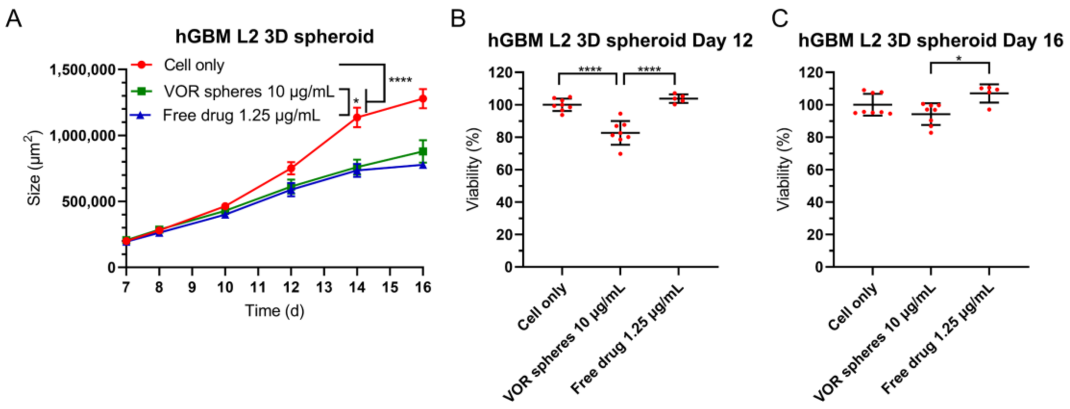

Figure S13. Vortioxetine microspheres reduce the size of the pre-established (grown) hGBM L2 3D spheroids. **(A)** shows the size changes during the treatment. Asterisks represent the statistically significant on Day 16 (Two-way ANOVA test, cell only and vortioxetine microspheres: n=8; free drug: n=5). **(B)** and **(C)** show the cell viability on Day 12 and Day 16, respectively. **(B)**: Ordinary one-way ANOVA test. **(C)**: Nonparametric Kruskal-Wallis test. For * $p\leq0.05$, **** $p\leq0.0001$. PLGA microspheres were added on Day 7. Abbreviation: VOR: vortioxetine. Error bars represent the mean ± SD.

Table S1. IC_{50} values of free temozolomide on hGBM cells and human astrocytes (µg/mL)

	**hGBM L0**	**hGBM L1**	**hGBM L2**	**hAstrocytes**
Day 2	198.8	71.37	89.34	853.1
Day 5	23.09	18.21	26.78	311.9
Day 8	7.82	28.24	32.33	59.19
